# Implications of the Wnt5a/CaMKII Pathway in Retinoic Acid-Induced Myogenic Tongue Abnormalities of Developing Mice

**DOI:** 10.1038/srep06082

**Published:** 2014-08-15

**Authors:** Wei Cong, Bo Liu, Shuqing Liu, Mingzhong Sun, Han Liu, Yue Yang, Ru Wang, Jing Xiao

**Affiliations:** 1 Department of Oral Pathology, College of Stomatology, Dalian Medical University, Dalian, Liaoning, 116044, China; 2 Department of Biochemistry, Dalian Medical University, Dalian, Liaoning, 116044, China; 3 Department of Biotechnology, Dalian Medical University, Dalian, Liaoning, 116044, China; 4 Department of Stomatology, the First Affiliated Hospital, Dalian Medical University, Dalian, Liaoning, 116011, China

## Abstract

Although proper tongue development is relevant to other structures in the craniofacial region, the molecular details of muscle development in tongue remain poorly understood. Here, we report that pregnant mice treated with retinoic acid (+RA) produce embryos with tongue malformation and a cleft palate. Histological analyses revealed that at E14.5, the tongues of +RA fetuses failed to descend and flatten. Ultrastructural analysis showed that at perinatal stage E18.5, the myofilaments failed to form normal structures of sarcomeres, and arranged disorderly in the genioglossus. The proliferation and levels of myogenic determination markers (Myf5 and MyoD) and myosin in the genioglossus were profoundly reduced. Wnt5a and Camk2d expressions were down-regulated, while levels of Tbx1, Ror2, and PKCδ were up-regulated in the tongues of +RA fetuses. In mock- and Wnt5a-transfected C2C12 (Wnt5a-C2C12) cells, Wnt5a overexpression impaired proliferation, and maintained Myf5 at a relative high level after RA treatment. Furthermore, Wnt5a overexpression positively correlated with levels of Camk2d and Ror2 in C2C12 cells after RA exposure. These data support the hypothesis that the Wnt5a/CaMKII pathway is directly involved in RA-induced hypoplasia and disorder of tongue muscles.

Proper tongue development is important to related structures in the craniofacial region; normal contractions of the tongue and other facial muscles control the forward growth of bone, cartilage growth and facial muscle bulk^1^. Although the core myogenic regulators including MyoD, Myf5, myogenin and MRF4 are required for tongue (somite-derived muscles) determination^2^,^3^,^4^, embryonic tongue muscles have unique characteristics that are distinct from other skeletal muscles such as limb and trunk^2^,^3^. In this context, the molecular details of muscle development in mammalian tongue remain poorly understood.

Retinoic acid (RA), a metabolite of vitamin A, is required for growth and development in chordate animals, including mice. RA provides intercellular signals that guide development *via* interactions with Hox genes^5^,^6^, regulate morphogenesis, cell proliferation and differentiation, and extracellular matrix production^7^,^8^. However, overdoses of RA, which are highly teratogenic, produce abnormalities in organ development. Exposure of pregnant mice to excess RA at a specific embryonic stage produces fetuses with cleft palate^9^. Our previous studies indicated that excess RA suppressed embryonic palatal mesenchymal cells proliferation during early development^10^. More interestingly, this peculiar type of abnormal tongue development also appears in RA-induced cleft palate mouse models. We also reported that Wnt5a- (a member of non-canonical Wnt pathway) deficient mice developed cleft palate and abnormal tongue distortion^11^. Thus, Wnt5a-regulated pathways might be involved in RA-induced tongue malformation in developing mice.

Concurrently, reduced RA synthesis, loss of Wnt5a or Tbx1 (a member of T-box transcription factors) led to similar phenotypes with cardiac abnormalities, i.e., severe hypoplasia of second heart field (SHF)-derived heart^12^,^13^. Increased Wnt5a expression was also found in perioptic mesenchyme of the eyes of RA-deficient mice^14^. Wnt5a also participates in myogenesis during embryonic development and activates myogenic determination in paraxial mesoderm of mouse embryos^15^. It is highly relevant that Wnt5a also plays a role in tongue size, fungiform papilla patterning and development through interaction with the Ror2 receptor^16^.

However, the aberrant morphogenesis and the molecular mechanisms that regulate myogenic development in the tongues of developing mice exposed to high-dose RA remain unclear. Here, we show that the Wnt5a/CaMKII pathway is implicated in RA-induced abnormal tongue myogenic development in embryonic mice. At E14.5 stage, myogenic cell proliferation in genioglossus is reduced, accompanied by down-regulations of Myf5 and MyoD. Subsequently at E18.5 stage, myofilaments fail to form normal sarcomere structures and were disordered arranged in the genioglossus. Compared to the fetal mice from control pregnant mice without RA treatment, Wnt5a was positively correlated with Camk2d level and inversely correlated with the levels of Tbx1, Ror2 and PKCδ in the tongues of fetal mice from pregnant mice exposed to high-dose RA. Using C2C12 cells, we further showed that stable expression of Wnt5a is closely linked to the proliferation and differentiation C2C12 cells. The positive association of Wnt5a with Camk2d and Ror2 in C2C12 cells in response to RA stimulation support our *in vivo* finding and indicate a direct involvement of the Wnt5a/CaMKII pathway in RA-induced tongue malformation.

## Results

### Fetuses of retinoic acid-treated pregnant mice develop tongue malformation

**Figure 1:**
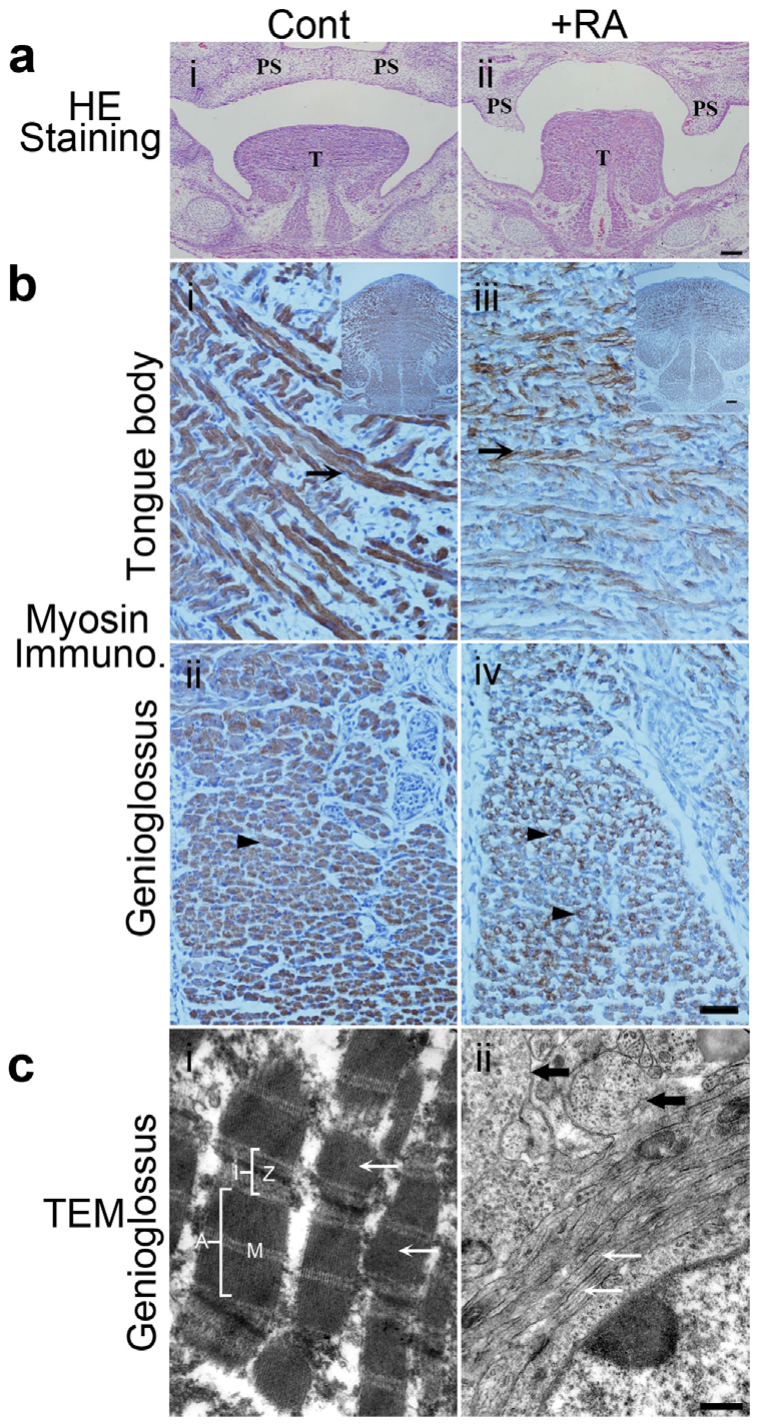
RA-induced tongue malformation at E14.5 and E15.5, and morphology of the genioglossus at E18.5. (a): HE staining of coronal sections of the tongue from E14.5 mouse fetus. The tongue (T) was at higher positions and bilateral palatal shelves (PS) were vertically positioned that formed a cleft in the +RA fetus (ii) versus control fetus (i). The bilateral palatal shelves (PS) in control fetus (i) already elevated above the tongue to the horizontal position and merged each other. Bar = 100 μm. (b): Myosin immunocytochemical staining of tongue muscles. Myosin expression in the tongue body (iii) and the genioglossus muscle (iv) were decreased in the +RA fetus versus control fetus. A higher magnification of the tongue intrinsic muscle from control fetuses showed multinucleated myotubes expressing high levels of myosin ((i), arrow). In +RA fetuses, myosin expression in myotubes ((iii) arrow) became weaker. In a transverse section of the genioglossus muscle, myosin was expressed at high levels in the control myotubes ((ii), arrowhead); in +RA fetuses, myosin staining was very weak. Bar = 40 μm. Upper right inserts in (i and iii) show images at lower magnifications, Bar = 100 μm. (c): Transmission electron microscopy examination of sagittal sections of the genioglossus. In control (i), myofibrils (white arrow) and sarcomeres were arranged longitudinally with integral I and A bands, and clear Z- and M-lines. In +RA (ii), the classic structures of sarcomeres of myofibrils were not found, myofilament bundles were arranged longitudinally (white arrow) and transversely (black arrow) in a single cell. Bar = 500 nm. Cont.: control mouse fetus; +RA: RA-exposed mouse fetus.PS: palatal shelves; T: tongue.

Morphology and ultrastructure of the tongues of E14.5, E15.5 and E18.5 mouse fetuses were examined by hematoxylin and eosin (HE) staining, immunohistochemical staining and transmission electron microscopy (Figure 1). At E14.5, fetal mice exposed to excess RA developed tongue deformities (Figure 1a). The tongues of control mouse fetuses were flat, and descended as a result of genioglossus muscle contraction. In addition, the bilateral palatal shelves moved upward, growing horizontally and maintained contact with the tongue (Figure 1a i). By contrast, the tongues of RA-exposed fetuses remained at a higher position, a consequence of failed flattening and descent (Figure 1a ii). Moreover, the bilateral palatal shelves extended vertically along both sides of the tongue, forming a cleft (Figure 1a ii).

We then examined tongue muscle development immunohistochemically, using myosin heavy chain as a marker of differentiation. At E15.5, a large number of myotubes were positive for myosin in both control and mutant group. Compared to fetuses from control pregnant mice (Figure 1b i and ii), the expressions of myosin in the tongue body and genioglossus were apparently decreased in RA-affected fetuses (Figure 1b iii and iv). At early stage of E14.5, only some myotubes were positive for myosin. In RA-affected fetuses, myosin staining in myotubes became weaker compared to control fetuses (Supplementary Figure 1).

According to transmission electron microscopy, the genioglossus of normal control mouse fetuses contained definitive sarcomere structures in myofibrils, i.e., complete light and dark bands, and clear Z-lines and M-lines (Figure 1c i), as well as the tongue body at E18.5 (Supplementary Figure 2); myofibrils were arranged orderly in the longitudinal (anteroposterior) direction (Figure 1c i). By contrast, significant defects including hypoplasia and muscle derangement were observed in genioglossus (Figure 1c ii), and hypoplasia in tongue body (Supplementary Figure 2) of mouse fetuses from RA-exposed pregnant females. In both of genioglossus (Figure 1c, Supplementary Figure 2) and tongue body (Supplementary Figure 2), RA exposure cause hypoplasia in the myocyte, only structures of myofilaments were detected, while these myofilaments failed to form classic sarcomere structures. Furthermore, in the sagittal section of genioglossus that were anteroposteriorly arranged, we noted the presence of transverse myofilament bundles adjacent to the longitudinal ones within the same myocyte, a characteristic of muscle derangement (Figure 1c ii, black arrow). We also observed that a great amount of myofilament bundles were arranged transversely among obliquely arranged ones (Supplementary Figure 2) in sagittal sections of genioglossus of +RA fetuses.

Thus, excessive RA exposure caused hypoplasia and disordered arrangements in tongue, especially in the genioglossus muscle, the major muscle responsible for tongue descent, resulting in high position of tongue.

### Retinoic acid inhibits proliferation and myogenic determination of tongue muscle

**Figure 2:**
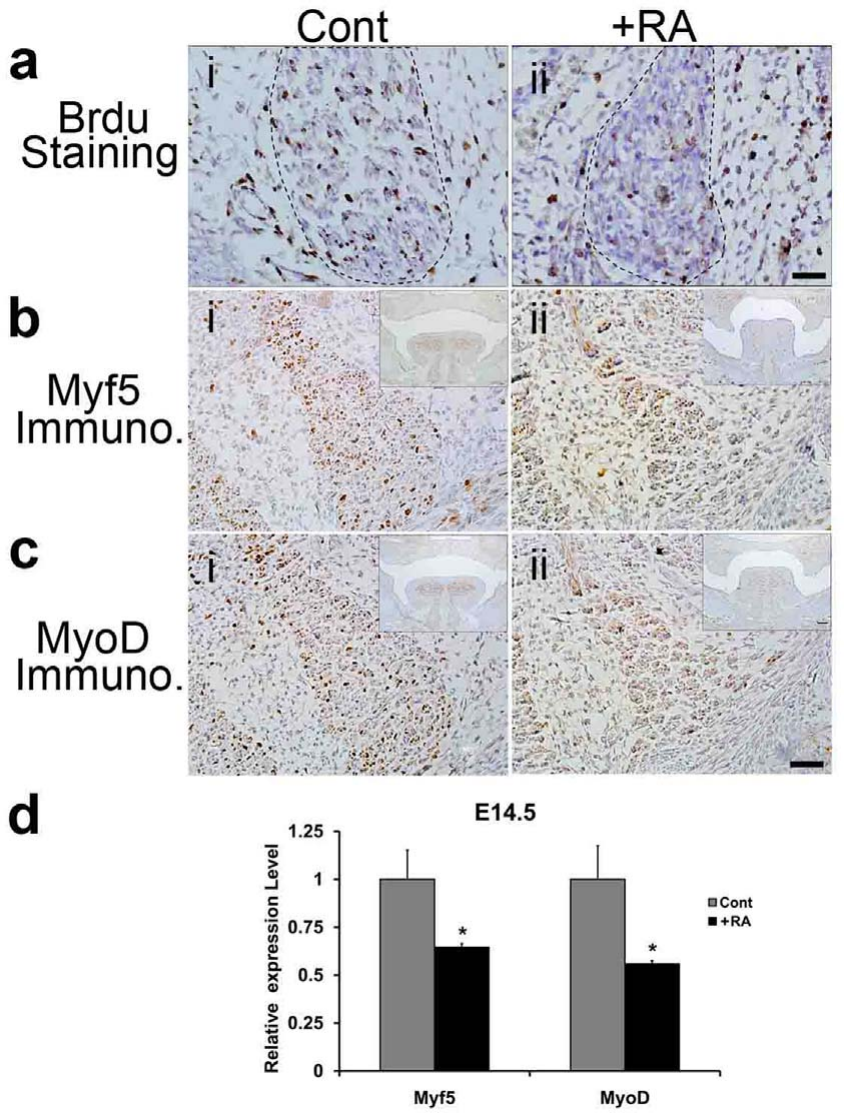
BrdU cell proliferation assay and immunohistochemistry (IHC) assay of the genioglossus at E14.5, and qRT-PCR analyses of Myf5 and MyoD expression at E14.5. (a): Cell proliferation assay. At E14.5, the numbers of BrdU positive cells/cm^2^ in the genioglossus of +RA mouse fetuses (ii), decreased to 35.3% of those observed in controls (i) (n = 3 mice per group, *P* < 0.01). The boundary of genioglossus was delineated by the dotted line. (b)–(c): IHC assay. (b) Myf5 protein level was apparently lower in the genioglossus of the +RA mouse fetus (ii) than control (i). Bar = 40 μm. (c) MyoD protein level in the genioglossus of the +RA fetus (ii) was lower than control (i). Bar = 40 μm. Upper right inserts in (b ii and c iii) show images at lower magnifications, Bar = 100 μm. (d) qRT-PCR analyses. The mRNA levels of Myf5 and MyoD decreased significantly in the +RA fetus as compared to the controls (n = 5, *P* < 0.05). Cont: control mouse fetus; +RA: RA-exposed mouse fetus.

Next, we determined if excess RA affects cell proliferation and myogenic determination of fetal tongue muscles. We measured the cell proliferation in the genioglossus of mouse fetuses collected from control and RA-exposed pregnant mice. At E14.5, the numbers of BrdU-staining positive cells/cm^2^ in genioglossus of mouse fetuses exposed to RA (Figure 2a ii) were 35.3% of those observed in control fetuses (Figure 2a i, *P* < 0.01). To identify if RA exposure impairs myogenic determination in fetal tongue, we measured mRNA and protein levels of Myf5 and MyoD, recognized early myogenic markers, in the tongues of mouse fetuses exposed to excess RA or vehicle alone. Myf5 and MyoD proteins were readily detected in the genioglossus of control group (Figures 2b i and 2c i), while in RA-treated group (Figures 2b ii and 2c ii), their corresponding levels were apparently lower than control. qRT-PCR analyses revealed that RA exposure decreased the mRNA levels of Myf5 (64.6%, *P* < 0.05) and MyoD (55.8%, *P* < 0.05) (Figure 2d).

### Wn5a/CaMKII pathway is involved in RA-induced tongue malformation in fetal mice

**Figure 3:**
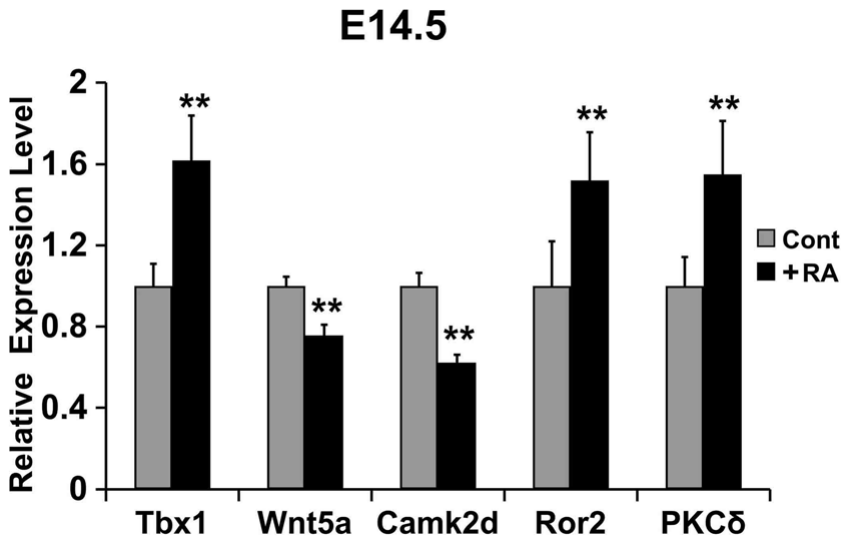
qRT-PCR analyses of the mRNA levels of Tbx1, Wnt5a, Camk2d, Ror2 and PKCδ in the tongues of E14.5 mouse fetuses. The mRNA levels of Tbx1, Ror2 and PKCδ in the +RA fetuses increased 62%, 52% and 55%, respectively, as compared to controls; the mRNA levels of Wnt5a and Camk2d in the +RA fetuses decreased 24% and 38%, respectively, compared to controls. Experiments were performed in triplicate, and ** denotes *P* < 0.01.

To determine the molecular mechanisms underlying RA-induced tongue malformation, we examined the expression profiles of Wnt5a, Camk2d, Ror2, Tbx-1 and PKCδ in the tongues of fetal mice using qRT-PCR. Compared to control fetal mice at E14.5, excess RA exposure decreased the mRNA levels of Wnt5a and Camk2d by 24% (*P* < 0.01) and 38% (*P* < 0.01), respectively, and up-regulated the mRNA levels of Ror2 (52%, *P* < 0.01), Tbx1 (62%, *P* < 0.01), and PKC-δ (55%, *P* < 0.01) (Figure 3). Thus, Wnt5a expression is positively correlated with Camk2d and negatively correlated with Ror2, Tbx1 and PKCδ expressions in the tongues of fetal mice exposed to excess RA.

### *In vitro* evidence for crosstalk between Wnt5a and Camk2d in mediating the effects of RA on myogenic development of tongue muscles

**Figure 4:**
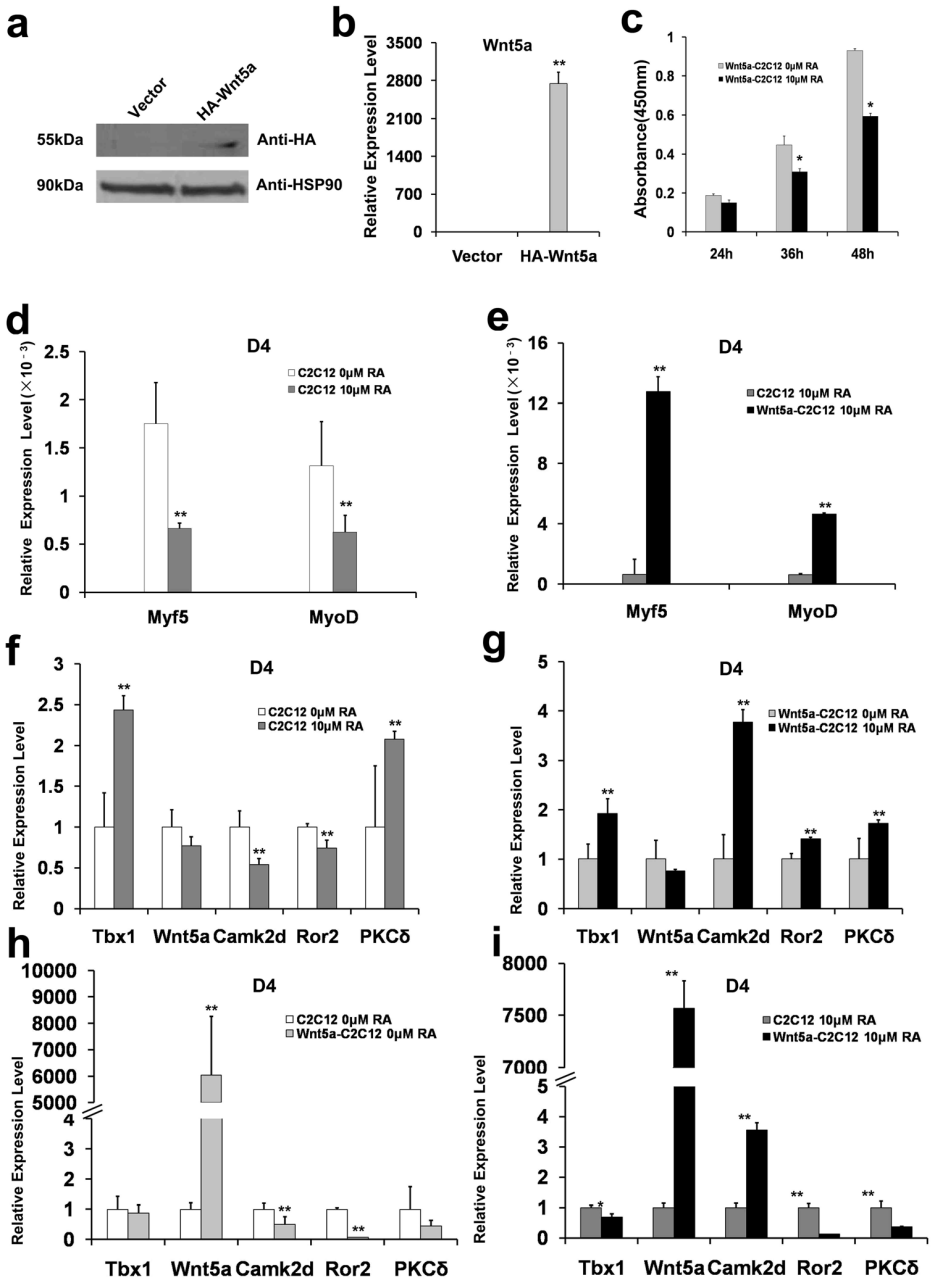
Western blot and qRT-PCR analyses of Wnt5a expression, and qRT-PCR analyses of Myf5, MyoD and related genes expression in differentiated C2C12 cells. (a). Western blot analysis of HA-Wnt5a expression. C2C12 cells were transfected with empty vector (Vector) or expression vector encoding the HA-tagged Wnt5a (HA-Wnt5a). HSP90 was used as a sample loading control. (b) qRT-PCR analysis of the mRNA level of HA-tagged Wnt5a. (c) Proliferation of HA-Wnt5a-C2C12 Cells with and without RA treatment. Following the indicated RA treatments, optical density at 450 nm of the HA-Wnt5a-C2C12 cells decreased to 80%, 69% and 63% of the control cells, at the time intervals of 24 h, 36 h, and 48 h, respectively. The changes at 36 h and 48 h were statistically significant (n = 3, *P < 0.05). (d) Differentiated C2C12 cells were treated with or without RA (10 μM), and the mRNA levels of Myf5 and MyoD were determined by qRT-PCR at day 4 (D4). (e) Differentiated control C2C12 cells and HA-Wnt5a-C2C12 cells were treated with 10 μM RA, and the mRNA levels of Myf5 and MyoD were determined using qRT-PCR (D4). (f) Differentiated C2C12 cells were incubated with or without RA (10 μM), and the mRNA levels of Tbx1, Wnt5a, Camk2d, Ror2 and PKCδ were determined by qRT-PCR (D4). (g) Differentiated HA-Wnt5a C2C12 cells were treated with or without RA (10 μM), and the mRNA levels of Tbx1, Wnt5a, Camk2d, Ror2 and PKCδ were measured using qRT-PCR (D4). (h)–(i): qRT-PCR analyses of expression levels of Tbx1, Wnt5a, Cam2d, Ror2, and PKCδ in control C2C12 cells and HA-Wnt5a C2C12 cells (D4) under basal condition (h) and after RA (10 μM) treatment (i). All experiments were performed in triplicate, and ** denotes *P* < 0.01. In (a), the full length blots were presented in Supplementary Figure S3.

C2C12 cell, originally derived from myoblast cell clones of adult C3H mouse leg muscle^17^, is an established skeletal muscle progenitor cell line that provides an ideal model system for studying skeletal muscle differentiation *in vitro*^18^. Since Wnt5a expressed at low level in C2C12 cells (unshown data), we established a stable C2C12 cell line expressing HA-tagged Wnt5 (HA-Wnt5a-C2C12 cells) using retroviral mediated gene delivery. Immunoblotting (Figure 4a) and qRT-PCR (Figure 4b) confirmed the overexpression of Wnt5a in HA-Wnt5a-C2C12 cells.

RA does not affect the proliferation (the Optical density at 450 nm) of wild-type C2C12 cells, as measured by the CCK-8 assay (data not shown). By contrast, when HA-Wnt5a C2C12 cells were treated with RA (10 μM) for 24 h, 36 h, and 48 h, the cell proliferation decreased by 20% (*P* = 0.057), 21% (*P* < 0.05) and 27% (*P* < 0.05), respectively, compared to HA-Wnt5a-C2C12 cells treated with vehicle alone (Figure 4c). Thus, the level of Wnt5a is associated with HA-Wnt5a-C2C12 cell proliferation in response to RA.

The mRNAs of Myf5 and MyoD in differentiated control- and HA-Wnt5a-C2C12 cells were readily detected (Figure 4d and Figure 4e). Following RA (10 μM) treatment, the mRNA levels of Myf5 and MyoD in control C2C12 cells decreased 62.2% and 52.5% (Figure 4d, *P* < 0.01), respectively,indicating the suppressed effect of RA on myogenic determination. By contrast, their corresponding levels in HA-Wnt5a-C2C12 cells were increased 19-fold and 7-fold by RA treatment (*P* < 0.01, Figure 4e) compared to vehicle treated Wnt5a-C2C12 cells. Thus, overexpression of Wnt5a in C2C12 myoblasts partially blocked RA-induced down-regulation of Myf5 and MyoD in differentiated C2C12 cells. Our data indicate that Wnt5a antagonizes the inhibitory effects of RA on C2C12 differentiation.

Next, we determined if RA treatment also affects the mRNA levels of Wnt5a, Tbx1, Camk2d, Ror2 and PKCδ in control and HA-Wnt5a-C2C12 cells. In differentiated control C2C12 cells, RA treatment (10 μM, 96 h), increased the mRNA levels of Tbx1 (240%, *P* < 0.01), Wnt5a (78%), Camk2d (50%), Ror2 (70%) and PKCδ (200%) (Figure 4f). Similarly, RA up-regulated the mRNA levels of these genes in differentiated HA-Wnt5a-C2C12 cells (Tbx1, 200%; Wnt5a, 76%; Camk2d, 370%; Ror2, 140%; and PKCδ, 170%) (Figure 4g). Meanwhile, we observed significant inverse correlations between Wnt5 overexpression and the mRNA levels of Camk2d, Ror2 and PKCδ in HA-Wnt5a- C2C12 cells (Figure 4i). Thus, our data support direct relationships between Wnt5a and Camk2d, and Wnt5a and Ror2. This hypothesis was further supported by results shown in Figure 4i. Wnt5a remained at relatively high levels in HA-Wnt5a-C2C12 cells, even after RA treatment. Meanwhile, Camk2d mRNA was significantly higher in HA-Wnt5a-C2C12 cells compared to control C2C12 cells. However, RA treatment decreased Ror2 mRNA level in HA-Wnt5a-C2C12 cells to a greater extent than that observed in control C2C12 cells (Figure 4i), its level was about 2-fold of its change in RA-treated- C2C12 against C2C12 cells (Figures 4 h and i). Similarly, the mRNA levels of Tbx1 and PKCδ were slightly more down-regulated in RA-treated HA-Wnt5a-C2C12 cells than their expression changes in HA-Wnt5a-C2C12 cells against C2C12 cells (Figures 4 h and i). Thus, the implicated inverse relationships between Wnt5a and Tbx1, and Wnt5a and PKCδ in this process require further investigation.

## Discussion

Normal tongue development requires contractions and homing of the tongue and other facial muscles^1^. Early movement of mouse tongue is adapted to functional activities such as suckling, swallowing, and chewing^1^. Prenatal exposure to excess RA triggers cleft palate formation in mouse fetuses^9^,^19^,^20^, while the malformation of tongue was rarely reported^21^,^22^. The aberrant morphogenesis of tongue and underlying molecular and cellular mechanisms that regulate tongue development in the presence of excess RA in fetal mice remain unclear. In current work, we also observed that the excessive RA exposure during pregnancy led to the failure of flattening and descent of tongue in mouse fetuses (Figure 1a ii), which is associated with defective subcellular changes of the tongue muscle including the hypoplasia and deranged tongue muscles, at E18.5 (Figure 1c ii).

How does excess RA induce tongue malformation in fetal mice? RA plays important roles in mediating germ cell development and neural differentiation of embryonic stem cells^23^,^24^,^25^. RA activates myogenesis by up-regulating myogenic markers MyoD and myogenin in somites, and myf5 in presomitic and somitic mesoderm in developing zebra fish embryos^26^. According to our data, at E14.5, the proliferations of genioglossus cells were greatly suppressed (Figure 2a) by excess RA. Based on IHC and qRT-PCR assays of the tongues of RA-exposed fetal mice at E14.5, which showed the down-regulation of Myf5 and MyoD, two myogenic determination markers (Figures 2b, c and d), we concluded that RA interferes with tongue myogenic differentiation. Genioglossus is the major muscle involved in tongue descent in fetal mice. Fetal tongue descent occurs before E14.5, indicating that muscle fibers are already mature and can contract in the genioglossus. At E18.5, failure of myofilaments to assemble into normal sarcomeres and derangement in the genioglossus of fetal mice lead to tongue malformation.

Wnt5a is involved in the developments of face, ears, genitals, limbs, distal digits^27^, and early embryonic myogenesis in mice^15^. We previously reported that Wnt5a-deficient mice developed cleft palate and distorted tongue^11^. In addition, the serine-threonine Ca^2+^/calmodulin-dependent protein kinase II (CaMKII)^28^ emulates the effects of Wnt5a during the myogenic development of chick wing bud, and is involved in Wnt5a-induced myogenic determination^29^. Loss of Wnt5a, or loss of Tbx1 together with reduced RA synthesis, results in cardiac hypoplasia^12^,^13^, so that Wnt5a may control tongue size, fungiform papilla patterning and development through interacting with the Ror2 receptor^16^. Based on the studies, we selectively examined the expression levels of Wnt5a, Camk2d, Ror2, Tbx1 and PKCδ in the tongues of fetal mice with or without RA exposure. At E14.5, RA exposure decreased Wnt5a mRNA level in the tongues of fetal mice, while reducing Camk2d mRNA and increasing Ror2, Tbx1 and PKCδ mRNA levels (Figure 3).

We also show that Wnt5a is involved in the RA-mediated inhibition of proliferation and differentiation of C2C12 cells. Since Wnt5a protein is not detected in C2C12 cells, and the growth of C2C12 cells is unaffected by RA (10 μM, data not shown), we established a stable C2C12 cell line over-expressing HA-tagged Wnt5a. RA decreased the rate of cell proliferation in HA-Wnt5a-C2C12 cells in a time-dependent manner (Figure 4b and Figure 4c), and decreased mRNA levels of Myf5 and MyoD (Figure 4d). Notably, Wnt5a over-expression markedly increased the levels of Myf5 and MyoD mRNA following RA exposure as compared to control cells (Figure 4e), indicating that Wnt5a antagonizes the suppressive effects RA on Myf5 and MyoD expression.

Our study also suggests a crosstalk between Wnt5a and Camk2d in tongue myogenic development following RA exposure. RA-induced down-regulation of Wnt5a mRNA in C2C12 cells was associated with reductions of Camk2d and Ror2 mRNAs, and up-regulations of Tbx1 and PKCδ mRNAs (Figure 4f). In HA-Wnt5a- C2C12 cells, however, RA-mediated suppression of Wnt5a mRNA level was associated with up-regulations of Tbx1, Camk2d, Ror2 and PKCδ (Figure 4g). Moreover, Wnt5a over-expression down-regulated the basal mRNA levels of Camk2d, Ror2 and PKCδ as compared to wild-type C2C12 cells (Figure 4h). The relative high levels of Wnt5a in HA-Wnt5a- C2C12 cells (Figure 4h, i), with or without RA treatment (10 μM), allowed us to observe a positive correlation between Wnt5a and Camk2d, and inverse correlations of Wnt5a with Tbx1, Ror2 and PKCδ. Based on the observation that RA-induced greater changes (2-fold) in Ror2 mRNA level in HA-Wnt5a-C2C12 cells compared to wild-type C2C12 cells (Figure 4h, Figure 4i), we infer a direct association of Wnt5a with Camk2d and Ror2 expression. The concomitant up-regulations of Wnt5a, Camk2d, and Ror2 might promote normal development of genioglossus cells by antagonizing the effect of excess RA. The potential inverse correlations of Wnt5a mRNA level with Tbx1 and PKCδ mRNAs in this process requires further investigation.

To sum up, our work shows that excess RA induces tongue malformation in fetal mice. At E14.5, tongues of fetal mice from pregnant females treated with RA failed to descend and flatten. Myofilaments of the genioglossus and tongue body failed to form normal structures of sarcomeres, resulting in hypoplasia of muscle fibers. Thus, the contraction force of genioglossus muscles will be decreased. Meanwhile, in genioglossus, a great amount of myofilament bundles ranged transversely in anteroposteriorly arranged ones, resulting in derangement in muscle fibers. So, the contracting directions of mutant genioglossus muscles will be inconsistent. RA exposure also impaired proliferation, determination and differentiation of the genioglossus cells. Taken together, the contraction effects of genioglossus will surely be decreased, and then cause high position of tongue. In vitro, Myf5 and MyoD were markedly down-regulated by RA in the genioglossus of fetal mice and in cultured C2C12 cells. Wnt5a plays a potentially important role in the normal development of mouse tongue, and regulates the rate of proliferation in C2C12 cells. Over-expression of Wnt5a antagonized RA-mediated down-regulation of Myf5 and MyoD in C2C12 cells. Results from both *in vivo* and *in vitro* studies indicate a crosstalk between Wnt5a, Camk2d, and Ror2 in myogenic development of the tongue in response to RA. Wnt5a/CaMKII pathway is involved in RA-induced abnormal myogenic development of tongue in fetal mice. While the potential negative correlation of Wnt5a with Tbx1 and PKCδ in tongue malformation following RA exposure needs further investigation, our study provides novel insights into RA-mediated birth defects and tongue-related diseases.

## Methods

### Animal experiment

Female ICR mice (10 ~ 12-week-old) were crossed with fertile males (ICR) overnight; the appearance of a vaginal plug was designated as GD 0.5 (gestation day 0.5). Pregnant mice at GD 10 were randomly divided into treatment and control groups, and respectively given all-trans retinoic acid (RA, 100 mg/kg) dissolved in edible oil, and edible oil only by gavage^10^. Pregnant mice were euthanized at E14.5, E15.5, or E18.5.

### Histochemical staining

At embryonic day 14.5 (E14.5), the heads of mouse fetuses were fixed in 4% paraformaldehyde (PFA), embedded in paraffin, sliced into 5-μm sections and fixed onto polylysine-coated slides. Tissue sections were then stained with hematoxylin and eosin by standard procedures.

### Myf5, MyoD and myosin assays

Immunohistochemical (IHC) staining was conducted using 4-μm paraffin-embedded embryonic tissue slices fixed in PFA for 4 h at 4°C. The primary antibodies used were as follows: rabbit anti-human Myf5 (1:400, Santa Cruz Biotechnology, Santa Cruz, CA, USA), rabbit anti-mouse MyoD (1:20, abcam, Cambridge, MA, USA), and monoclonal mouse anti-myosin (1:100, Maixin, Fuzhou, China). Secondary antibody MaxVision™ HRP- Polymer anti-Mouse/Rabbit was from Maixin. Diaminobenzidine (DAB, Maixin, Fuzhou, China) was used as chromogen for color development and hematoxylin was used for counterstain.

### Transmission electron microscopy (TEM)

Muscle samples from the tongue body and genioglossus at E18.5 were immediately fixed in 2.5% glutaraldehyde for 2 h, post-fixed with 2% osmium tetroxide for 2 h, deyhdrated in a graded ethanol series, and embedded in Epon 812. The genioglossus was sectioned sagittally, and the tongue body was sectioned horizontally. Ultrathin sections were obtained using an Ultramicrotome (Leica EM UC6, Germany). All sections, mounted on copper grids, were contrasted using uranyl acetate and lead citrate before being viewed using a transmission electron microscope (JEM-2000EX).

### Cell proliferation assay

Pregnant mice at GD14.5 were injected intraperitoneally with BrdU (100 mg/kg, Sigma, St. Louis, MO, USA). After 20 min, mouse fetuses were collected. Sections of the fetal tongue muscles were treated with 3% H_2_O_2_ for 10 min, 0.1% trypsin for 6 min, and HCl (2 mol/L) for 30 min to denature DNA, and then incubated with biotinylated mouse anti-BrdU monoclonal antibody (1:500, Sigma, St. Louis, MO, USA) overnight at 4°C, and MaxVision™ HRP-Polymer anti-Mouse/Rabbit polyantibody (Maixin, Fuzhou, China) for 15 min. DAB was used as a chromogen for color development and hematoxylin as a counterstain.

### RNA extraction, reverse transcription, and quantitative real-time PCR (qRT-PCR)

Total RNA was extracted from fetal tongue muscles using Trizol (Invitrogen, Grand Island, NY, USA). PrimeScript RT reagent Kit and gDNA Eraser kit (Takara, Japan) were used for the reverse transcriptions of mRNA and removal of genomic DNA. cDNA was synthesized at 37°C for 15 min and 85°C for 5 sec using a MyCycler™ Thermal Cycle system (Bio-Rad, Hercules, CA, USA).

Complete mRNA sequences of Myf5 (NM_008656.5), MyoD (NM_010866.2), Tbx1 (NM_ 011532.1), Wnt5a (NM_009524.2), Camk2d (NM_ 001025438.1), Ror2 (NM_ 13846.3) and PKCδ (NM_008857.3) were retrieved from GenBank. GAPDH was used as a sample loading control (Table 1). qRT-PCR was performed using SYBR Premix Ex Taq™ II kit in a DiceReal Time System Thermal Cycler (TP800, Takara, Japan). Reactions were performed in 25-μL containing cDNA (2.0 μL), SYBR Premix Ex Taq™ II (2×, 12.5 μL), PCR forward Primer (1.0 μL), PCR reverse primer (1.0 μL), and ddH_2_O (8.5 μL). PCR was performed at 95°C for 10 sec, followed by 40 cycles of 95°C for 5 sec and 60°C for 30 sec.

### Cell culture

The murine skeletal muscle cell line C2C12 was purchased from the cell bank of Type Culture Collection of The Chinese Academy of Science (Shanghai, China). C2C12 myoblasts were cultured in high-glucose Dulbecco's modified Eagle's medium (DMEM, Invitrogen, USA) supplemented with 10% fetal bovine serum (FBS, Invitrogen, USA) at 37°C with 5% CO_2_ as previously described^30^. At 48 h, C2C12 myoblasts (~80% confluent) were induced to differentiate using high-glucose DMEM supplemented with 2% horse serum (Hyclone, USA) and treated immediately with RA (10 μM final concentration, Sigma-Aldrich, USA) dissolved in dimethyl sulfoxide (DMSO) for experimental group cells and with DMSO for control group cells. Media containing fresh RA were changed every 2 days for experimental group cells. Cells were collected 4 days post induction of differentiation. RNA extraction and qRT-PCR were performed as described above.

### Wnt5a overexpression

Plasmid construction and viral infection. Plasmids were constructed using standard methods; sequences were verified by restriction digestion and/or sequencing. Mouse full-length *Wnt5a* cDNA fused with hemagglutinin (HA) tag was cloned from total RNAs extracted from C2C12 cells using standard PCR protocol. HA-*Wnt5a* sequence was subcloned into the pLNCX retroviral vector. For viral packaging, 3 × 10^6^ 293T cells were incubated in a 10-cm tissue culture plate containing 10 mL DMEM + 10% FBS without antibiotics overnight. Retroviral plasmid (2 μg), packaging plasmid (2 μg), envelope plasmid (2 μg), and FuGENE® 6 transfection reagent (18 μL; Promega, USA) were added to 600 μL serum-free OPTI-MEM (Invitrogen, USA); after a 30-min incubation at room temperature, the mixture was gently added to 293T cells dropwise. After 12 ~ 15 h, the transfection mixture was removed and cells were incubated in 10 mL of DMEM supplemented with 10% FBS, 100 U/mL penicillin, and 100 μg/mL streptomycin. Seventy-two hours later, the virus-containing media were harvested, centrifuged at 1, 250 rpm for 5 min and filtered through a 0.22 μm filter to remove cells. The collected viruses were stored at −80°C. For viral infections, cells plated the day before infection were incubated with media containing appropriate amount of retroviruses in the presence of polybrene (6 μg/ml, Sigma-Aldrich, USA) for 6 hrs. Forty-eight hours later, the infected cells were selected using G418 (600 μg/ml) for 14 days.

### Western blot analysis

Cells were lysed in RIPA buffer (10 mM Tris-HCl, 1 mM EDTA, 1% SDS, 1% NP-40, 1:100 protease inhibitor cocktail, 50 mM β-glycerophosphate, 50 mM sodium fluoride). Cell lysates were separated by 10% SDS-PAGE and transferred to PVDF membranes using semi-dry transfer apparatus (Bio-Rad, USA). The membranes were incubated with 5% non-fat dry milk for 2 h, and then with primary antibodies overnight, followed by HRP-conjugated anti-rabbit or anti- mouse IgG secondary anbitodies (Promega, USA), and visualized with SuperSignal reagents (Pierce, USA). Primary monoclonal antibody against HA (Clone No.C29F4, Cat No.3724, Cell Signaling Technology, Danvers, USA) was used. Polyclonal HSP90 antibody (Cat No.Sc-7947, Santa Cruz, USA) was used as an internal standard.

### Cell proliferation of C2C12 cells

C2C12 cells and Wnt5a-overexpressing C2C12 cells were incubated with or without RA for 24, 36 and 48 h. Cell proliferation was analyzed using Cell Counting Kit-8 (CCK-8, Dojin Laboratories, Kumamoto, Japan), according to manufacturer's instructions.

### Statistical analysis

IHC assays were performed in triplicate; data were analyzed using unpaired Student's t-test. For qRT-PCR, three independent biological samples were used to establish statistical significance, and the data were analyzed using two independent sample tests of double ΔCt values. Statistical analyses were conducted using SPSS 13.0 software, and were considered significant at *P* < 0.05.

### Ethics statement

All procedures using mice were approved by the Ethical Committee of the Dalian Medical University and performed under strict ethical guidelines (L2012013). All experiments using mice were performed in accordance with the guidelines of the Animal Management Committee of Dalian Medical University. Animals were housed at the Laboratory Animal Center of the Dalian Medical University (Animal license SYXK [Liao] 2008-0002), and had access to feed and water ad libitum. The facility, accessed by authorized personnel only, is controlled for temperature, ventilation and lighting.

## Author Contributions

J.X. designed the study. W.C. and Y.Y. performed immunohistochemical staining. W.C. and B.L. performed transmission electron microscopy experiments. B.L. performed PCR experiments. B.L. and H.L. prepared figures. L.S. and R.W. analyzed the data. C.W. and S.M. wrote the paper. All authors discussed the results and commented on the manuscript.

## Supplementary Material

Supplementary Information Supplementary Information

## Figures and Tables

**Table 1:** Designed Primer Sequences

Gene Tested	Accession No.	Primer Sequence
Myf5	NM_008656.5	F: TGAATGTAACAGCCCTGTCTGGTC
		R: CGTGATAGATAAGTCTGGAGCTGG
MyoD	NM_010866.2	F: CGCTCCAACTGCTCTGATGGCA
		R:TGCTGCTGCAGTCGATCTCTCA
Tbx1	NM_011532.1	F: CTTTCGACAAGCTGAAACTGACCA
		R: TTTCGAGGGTCCACATAGACAACA
Camk2d	NM_001025438.1	F: AGAAGTTCAAGGCGACCAGCA
		R: GGGTATCCCACCAGCAAGATGTAG
Wnt5a	NM_009524.2	F: AATCCACGCTAAGGGTTCCTATGAG
		R: AGCCAGCACGTCTTGAGGCTA
Ror2	NM_013846.3	F: CATTGGGAACCGGACTATTTATGTG
		R: CTGGTCTGACAGTTGCGTGGA
PKCδ	NM_008857.3	F: TCATCTGCGGACTGCAGTTTCTA
		R: CAAAGTCAGCGATCTTGATGTGG
GAPDH	NM_008084.2	F: TGTGTCCGTCGTGGATCTGA
		R: TTGCTGTTGAAGTCGCAGGAG

Note: F: foreword primer; R: reverse primer.
