# Supplementary material for: Implications of the Wnt5a/CaMKII Pathway in Retinoic Acid-Induced Myogenic Tongue Abnormalities of Developing Mice
Source: Sci Rep. 2014 Aug 15;4:6082. doi: 10.1038/srep06082 (PMC4133706; doi:10.1038/srep06082)
Supplement: Supplementary Information [file srep06082-s1.pdf]

Supplementary Information

Implications of the Wnt5a/CaMKII Pathway in Retinoic Acid-Induced Myogenic Tongue Abnormalities of Developing Mice

W Cong[1], B Liu[1], S Liu[2], M Sun[3], H Liu[1], Y Yang[1], R Wang[4] and J Xiao[1*]

Supplementary Figure S1

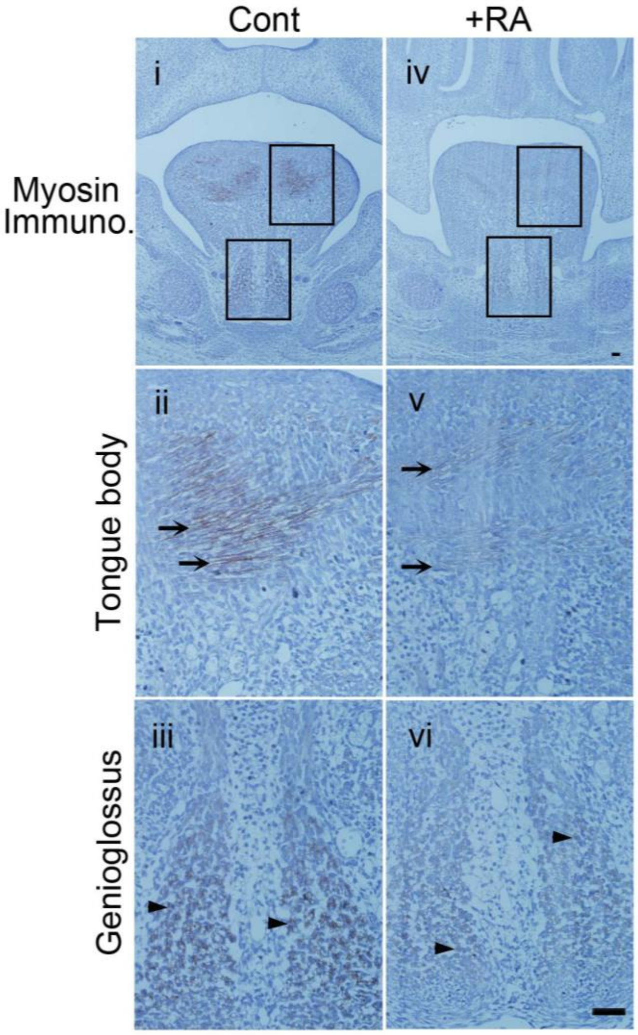

Supplementary Figure S1. Myosin immunocytochemical staining of tongue muscles at E14.5.

In crown sections of the heads, myosin expressions in the tongue intrinsic muscle and the genioglossus muscle were apparently decreased in the +RA fetus (**iv**) versus control fetus (**i**). Bar = 100 μm. A higher magnification of the tongue intrinsic muscle from control fetuses showed multinucleated myotubes expressing myosin (**ii**, arrow). In +RA fetuses, myosin expression in myotubes (**v**, arrow) became weaker. Myosin was expressed at high levels in the control myotubes (**iii,** arrowhead); in +RA fetuses, myosin staining was very weak (**vi**, arrowhead). Bar = 40 μm. Cont.: control mouse fetus; +RA: RA-exposed mouse fetus.

Supplementary Figure S2

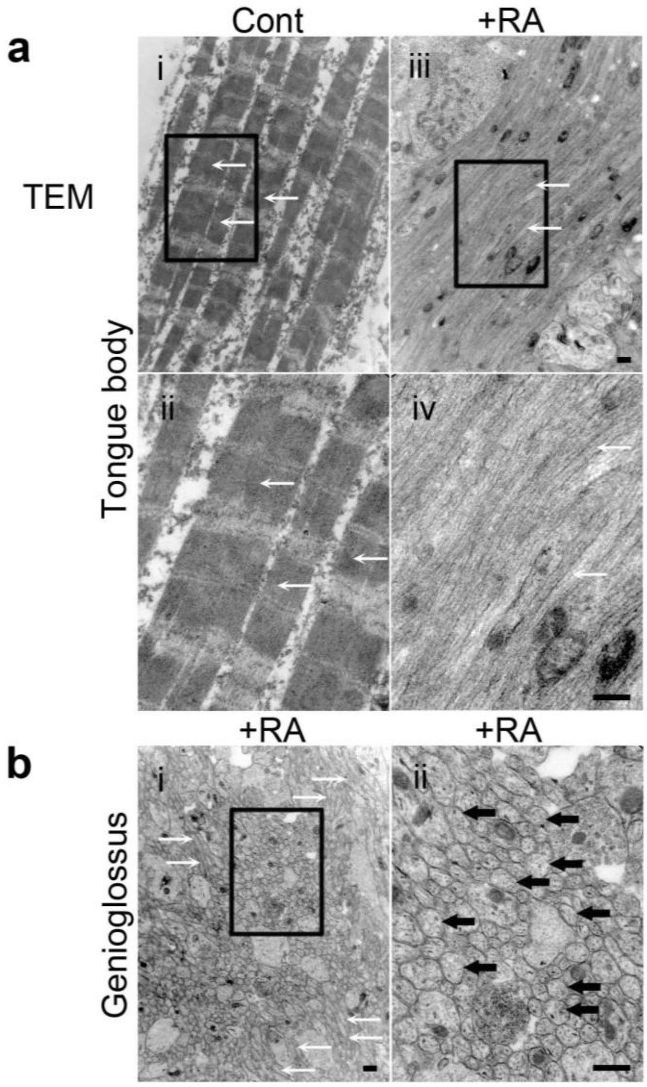

Supplementary Figure S2. Ultrastructural changes of fetal tongue by transmission electron microscopy examination at E18.5

(a): Transverse sections of the tongue body. In control (**i, ii**), myofibrils (white arrow) contained definitive sarcomere structures. In +RA **(iii, iv)**, the classic structures of sarcomeres of myofibrils were not found, only the structures of myofilaments (white arrow) can be detected. **(b):** Sagittal sections of the genioglossus in +RA fetus. (**i**, **ii**) A great amount of myofilament bundles were arranged transversely (black arrow) among obliquely arranged bundles (white arrow). Bar =

500 nm. Cont.: control mouse fetus; +RA: RA-exposed mouse fetus.

Supplementary Figure S3

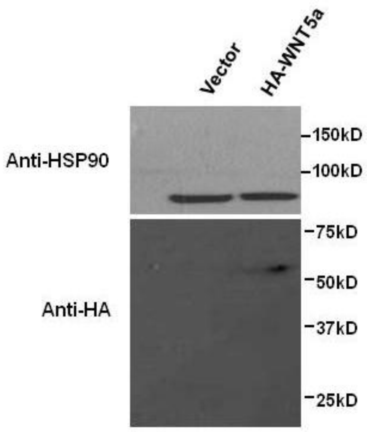

Supplementary Figure S3. Uncropped images of Western blot.
